# Ginsenoside Rd promotes glutamate clearance by up-regulating glial glutamate transporter GLT-1 via PI3K/AKT and ERK1/2 pathways

**DOI:** 10.3389/fphar.2013.00152

**Published:** 2013-12-11

**Authors:** Xiao Zhang, Ming Shi, Magnar Bjørås, Wei Wang, Guangyun Zhang, Junliang Han, Zhirong Liu, Yunxia Zhang, Bing Wang, Jing Chen, Yi Zhu, Lize Xiong, Gang Zhao

**Affiliations:** ^1^Department of Neurology, Xijing Hospital, The Forth Military Medical UniversityXi’an, Shaanxi, China; ^2^Department of Microbiology, Institute of Clinical Medicine, Oslo University HospitalOslo, Norway; ^3^Department of Anesthesiology, Xijing Hospital, The Forth Military Medical UniversityXi’an, Shaanxi, China

**Keywords:** ginsenoside Rd, GLT-1, astrocyte, glutamate, PI3K/AKT, ERK1/2

## Abstract

Ginsenoside Rd (Rd), one of the main active ingredients in *Panax ginseng*, has been showed to protect against ischemic cerebral damage both *in vitro* and *in vivo*. However, the underlying mechanism of Rd is largely unknown. Excessive extracellular glutamate causes excitatory toxicity, leading to cell death, and neurodegenerative processes after brain ischemia. The clearance of extracellular glutamate by astrocytic glutamate transporter GLT-1 is essential for neuronal survival after stroke. Here we investigated the effects of Rd on the levels of extracellular glutamate and the expression of GLT-1* in vivo* and *in vitro*. After rat middle cerebral artery occlusion, Rd significantly increased the mRNA and protein expression levels of GLT-1, and reduced the burst of glutamate as revealed by microdialysis. Consistently, specific glutamate uptake by cultured astrocytes was elevated after Rd exposure. Furthermore, we showed that Rd increased the levels of phosphorylated protein kinase B (PKB/Akt) and phospho-ERK1/2 (p-ERK1/2) in astrocyte culture after oxygen–glucose deprivation. Moreover, the effect of Rd on GLT-1 expression and glutamate uptake can be abolished by PI3K/AKT agonist LY294002 or ERK1/2 inhibitor PD98059. Taken together, our findings provide the first evidence that Rd can promote glutamate clearance by up-regulating GLT-1 expression through PI3K/AKT and ERK1/2 pathways.

## INTRODUCTION

In spite of advances in technology and improved clinical care, ischemic stroke still remains a major cause of mortality and disability in human and the second leading cause of death in developed countries (Goldstein et al., 2006). Two major approaches have been developed to treat acute ischemic stroke: recanalization and neuroprotection (Gropen et al., 2006). The goal of neuroprotective therapy is to save potentially viable brain tissue in the ischemic penumbra. Unfortunately, although many neuroprotective interventions are effective for stroke in the animal model, they failed to benefit to patients in clinical trials. Thus, developing effective drugs to treat acute ischemic stroke still faces challenges.

Ginseng, the root of *Panax ginseng* C. A. Meyer (Araliaceae), has been widely used as a kind of traditional Chinese herbal medicine for 1000s of years. Ginsenosides are the most active ingredients in ginseng. Up to now more than 40 different ginsenosides, including ginsenoside Rd (Rd) have been identified (Radad et al., 2006). Our randomized, double-blind, placebo-controlled, multicenter trial showed that Rd is effective and safe for the treatment of acute ischemic stroke (Liu et al., 2009, 2012b).

In our pre-clinical studies, we found that Rd can prevent glutamate/oxygen–glucose deprivation (OGD)-induced apoptosis in cultured neurons (Ye et al., 2009; Li et al., 2010), and reduce infarction volume after transient focal ischemia in rats (Ye et al., 2011a, b), suggesting that Rd can be served as a promising neuroprotectant. However, the underlying mechanisms of Rd neuroprotection are still not fully elucidated. Numerous approaches in neuroprotection have considered the application aimed at targeting non-neuronal cells (Van der Schyf et al., 2006). Besides directly supporting neurons, Rd can interfere with a number of other cells such as astrocytes (Lopez et al., 2007). Astrocytes play an important role in supporting neurons in physiological and pathological conditions by producing various growth factors. Particularly, they are the key cells for the uptake of excitatory neurotransmitter glutamate (Danbolt, 2001). Excessive extracellular glutamate elicits neurotoxicity and is mainly removed by glutamate transporters, GLAST (EAAT1) and GLT-1(EAAT2), exclusively expressed on astrocytes (Gegelashvili and Schousboe, 1997). GLAST predominantly expresses in the cerebellum and GLT-1 in the cerebral cortex and hippocampus (Danbolt, 2001). In the forebrain, more than 90% of the glutamate uptake is mediated by GLT-1(Danbolt, 2001), and dysfunction or knockout of GLT-1 gene leaded to elevation of extracellular glutamate and exacerbation of acute cortical injury (Rao et al., 2001; Mitani and Tanaka, 2003). Studies showed that GLT-1 expression was regulated by PI3K/AKT and ERK1/2 pathways (Li et al., 2006; Lee et al., 2012a) and ginsenosides can activate these pathways (Lan et al., 2011; Hashimoto et al., 2012; Yan et al., 2013). Therefore, in this study we investigated the effects of Rd on extracellular glutamate metabolism and the expression of GLT-1, and further explored whether PI3K/AKT and ERK1/2 pathways were involved in this process.

## MATERIALS AND METHODS

### MATERIALS

Rd with a purity of 98% was obtained from Tai-He Biopharmaceutical Co. Ltd. (Guangzhou, China). The stock solutions were prepared in saline containing 10% 1,3-propanediol (v/v). Hoechst 33342 was purchased from Sigma–Aldrich Inc. (St. Louis, MO, USA). The commercial kit for the detection of glutamate was purchased from CMA (Solna, Sweden). GLT-1 antibody was obtained from Abcam (Cambridge, UK) and other antibodies were purchased from Cell Signaling (Danvers, MA, USA). All other reagents were from commercial suppliers and of standard biochemical quality.

### FOCAL CEREBRAL ISCHEMIA

Male Sprague–Dawley rats weigh 270–320 g were used in this study. Animal protocols were approved by the Ethics Committee for Animal Experimentation of the Fourth Military Medical University. The focal cerebral ischemia was induced by 1.5 h of middle cerebral artery occlusion (MCAO) as described previously with modifications(Kramer et al., 2010). In brief, animals were anesthetized with a mixture of isoflurane (1.5–2%), oxygen and nitrogen. Body temperature in the rectum was maintained at 37°C using a thermostatically controlled heating blanket connected to a thermometer probe. A 4-0 nylon monofilament coated with poly-L-lysine was introduced through the internal carotid artery to occlude the origin of the middle cerebral artery (MCA). The induction of focal cerebral ischemia was verified with laser Doppler flowmetry (PeriFlux 5000; Perimed AB, Sweden). A drop in regional cerebral blood flow (CBF) below 30% from baseline after the insertion of the filament was considered to be sufficient for induction of focal cerebral ischemia. Control animals were subjected to the same surgical procedures except that the suture was not advanced into the MCA. Rd with a concentration of 30 mg/kg or vehicle was applied intraperitoneally 1 h before MCAO. Animals were sacrificed at designated times points after MCAO and samples were collected for Western blot and reverse transcription polymerase chain reaction (RT-PCR) experiments analysis. The tissues from the ischemic core and penumbra were dissected according to the protocol described by Kramer et al. (2010).

### ASTROCYTE CULTURES

Astrocytes were cultured from newborn Sprague–Dawley rats as previously described with some modifications (Lopez et al., 2007). Rat cortex and striatum were isolated and minced. After trypsinization, dissociated cells were passed through sterile nylon meshes, seeded to 75 cm^2^ flask at a density of 1 × 10^6^ cells and cultured in Dulbecco’s-modified eagle medium (DMEM, GIBCO) supplemented with 10% heat-inactivated fetal bovine serum at 37°C. The medium was renewed three times a week. Microglia and oligodendrocytes were removed by shaking at 260 pm overnight at 37°C in an orbital shaker (ShenTong, China). Astrocytes were collected and sub-cultured for 24 h before experiments.

### OXYGEN–GLUCOSE DEPRIVATION

Oxygen–glucose deprivation was carried out as described by Li et al. (2010). Briefly, the culture medium was replaced with pre-warmed DMEM without glucose and serum. The cell cultures were then transferred into an anaerobic chamber equilibrated with 95% N_2_ and 5% CO_2_. The chamber was kept in a 37°C incubator. Control cultures were maintained in a normal oxygenated DMEM containing 25 mM glucose. After 3 h, cultures were placed back to the normoxic incubator with normal culture medium.

### MICRODIALYSIS

Microdialysis was performed as described previously (Ye et al., 2011b). Briefly, a microdialysis probe (BAS MD-2204, 4 mm membrane) was stereotaxically inserted into the right striatum through the cannula guide (-3 mm anteroposterior, +4 mm mediolateral to the Bregma, -4 mm dorsoventral from the surface of the brain). Artificial cerebrospinal fluid (NaCl 135 mM, KCl 1 mM, CaCl_2_ 1.2 mM, MgCl_2_ 1 mM, pH 7.4) was perfused at 2 μl/min using a microinjection pump (BeeHive BAS, USA). The microdialysis samples were continuously collected into microvials collected every 20 min. Three samples were collected as baseline values at the end of 2 h equilibration period. The concentrations of glutamate in the microdialysis samples were determined using a CMA 600 Analyzer (Solna, Sweden). Level changes for all measured chemicals were expressed as percent relative to the mean baseline value.

### EVALUATION OF GLUTAMATE UPTAKE

Extracellular glutamate levels were measured by a fluorimetric method using the Amplex Red Glutamic Acid assay kit (Invitrogen) as described previously with some modifications (Sato et al., 2003; Pawlak et al., 2005). After 3 h of OGD astrocyte culture medium was replaced by Hepes buffer containing 25 mM glucose and 500 μM glutamate. At each time point, 50 μl of supernatants was transferred into 96-well microplates, and then mixed with 50 μl substrate mixture (100 mM Amplex Red, 0.25 U/ml horseradish peroxidase, 0.08 U/ml L-glutamate oxidase, 0.5 U/ml glutamate pyruvate transaminase, and 200 μl alanine) and incubated at 37°C for 30 min. Fluorescence was measured using an automated microplate reader at a wavelength of 530 nm (vs. reference wavelength of 590 nm). Glutamate concentrations were calculated from the standard curve with known glutamate amounts.

### IMMUNOCYTOCHEMISTRY

Immunofluorescence labeling was performed as described (Plachez et al., 2004). Cultured cells were fixed with 4% (w/v) paraformaldehyde for 20 min and then washed with phosphate buffered saline (PBS). The cells were permeablized with 0.1% Triton X-100 in PBS for 1 h at room temperature. After blocked with bovine serum albumin (BSA) for 30 min, the cultures were incubated with primary antibody (GLT-1 1:100) in PBS containing 0.5% (w/v) BSA and 0.1% triton X-100 at 4°C overnight. After washed with PBS for three times, the cells were incubated with biotinylated anti-rabbit IgG (1:200) for 2 h, followed by streptavidin-cy3(1:1000) for 2 h. Hoechst 33342(1 μg/ml) was used for counterstain. The immunostaining images were observed under a fluorescence microscope (Leica, Germany).

### REVERSE TRANSCRIPTION-POLYMERASE CHAIN REACTION

Brain tissues were collected at 24 h following MCAO. RT-PCR was performed as described (Pawlak et al., 2005). Total RNA of brain tissues was extracted using Trizol reagent (Invitrogen Life Technologies, Carlsbad, CA, USA) according to the manufacturer’s instructions. 3 μg of total RNA was used for reverse transcription using Revert Aid^TM^ First Strand cDNA Synthesis Kit (Fermentas, Burlington, ON, Canada). The PCR amplification was carried out as follows: a 30 min incubation at 58°C for cDNA synthesis, a 3 min hot start at 94°C followed by 32 cycles of denaturation at 94°C for 30 s, annealing at 56°C for 30 s, and extension at 72°C for 30 s with a final extension at 72°C for 5 min. The amplified products were electrophoretically separated by 1.5% agarose gels containing ethidium bromide. Data were normalized to β-actin expression. Forward/reverse primers were:

GLT-1 5′–CAAGCTGATGGTGGAGTTCTT-3′/5′-CACGCTTG TCAATCCCTAGAT-3′; β-actin 5′-TGTGGCATCCATGAAACTAC A-3′/5′-CCACCAATCCACACAGAGTAC-3′.

### WESTERN BLOT

Tissue of interest was homogenized on ice in the RIPA lysis buffer (Beyotime, China) containing 0.5 mM phenylmethylsulfonyl fluoride (PMSF). Western blot was performed according to Ye et al. (2011b). Briefly, protein samples were electrophoresed on a 10% SDS-PAGE and subsequently transferred to polyvinylidene difluoride (PVDF) membrane (Millipore, Billerica, MA, USA). The membrane was incubated in blocking buffer containing 5% non-fat dried milk at room temperature for 1 h and then probed with the primary antibody [GLT-1 at 1:1000; p-AKT at 1:500; p-ERK at 1:500; ERK at 1:1000; AKT at 1:1000; and glyceraldehyde 3-phosphate dehydrogenase (GAPDH) at 1:2000] in blocking buffer at 4°C overnight. The membrane was washed three times with TBST [tris-buffered saline (TBS) and 0.1% Tween 20] and then incubated with horseradish peroxidase (HRP)-conjugated secondary antibody at room temperature for another 1 h. Specific signals of proteins were visualized by chemiluminescence using the enhanced chemiluminescence (ECL) western blotting detection system (GE Healthcare, UK). For quantitative analysis, the ratio of the specific signals of protein (relative intensity of the signal) to that of GAPDH protein was calculated.

### STATISTICAL ANALYSIS

All results were presented as mean with standard error mean (SEM). Microdialysis and glutamate uptake results were analyzed with repeated-measures analysis of variance, followed by Tukey HSD *post hoc*. Other results were analyzed using one-way ANOVA followed by Tukey HSD *post test* for multiple comparisons. Originpro 8 software was used for statistical tests. Statistical significance was established at *p* < 0.05.

## RESULTS

### Rd PROMOTES GLUTAMATE CLEARANCE *IN VITRO* and *IN VIVO*

**FIGURE 1:**
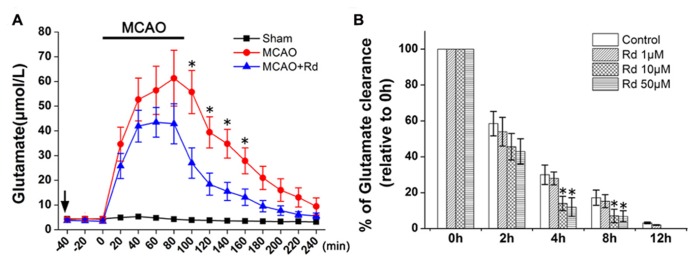
**Effect of Rd treatment on the extracellular concentrations of glutamate** **(A)** Time course of the glutamate concentration determined with microdialysis in MCAO rats (*n* = 6), MACO rats pre-treated with Rd (depicted as black arrow, 30 mg/kg, *n * = 8) or sham-operated rats (*n* = 7) subjected to 90-min MCAO (depicted as the black bar) followed by 150-min reperfusion. Values are the means ± SEM. *p<0.05 vs. MCAO+Rd group. **(B)** Glutamate uptake was measured by indirectly quantifying the clearance of extracellular glutamate added. After 3 h of OGD, 500 μM glutamate was added to culture medium. Glutamate concentrations were measured at different time points as indicated after OGD washout. Data are expressed as mean ± SEM (*n* = 6). Mean values of 500 μM glutamate were scaled to 100%.*p < 0.05 vs. control group.

To determine the effects of Rd on extracellular glutamate metabolism *in vivo*, we measured concentrations of extracellular glutamate during the period of 1.5 h ischemia and 2.5 h reperfusion in the ischemic striatum. As shown in Figure **1A**, MCAO induced a rapid and marked increase in levels of extracellular glutamate, which reached the maximum at 60–100 min after stroke onset. The concentration of glutamate decreased after reperfusion but did not return to the basal lines during the measurement period in MCAO group. Compared with MCAO group, Rd administration 1 h before MCAO attenuated glutamate burst and promoted its recovery to the baseline 150 min after reperfusion. The differences in glutamate levels between MCAO and Rd-treated rats were significant at 100, 120, 140, and 160 min of occlusion (**Figure 1A**).

Furthermore, the effect of Rd on astrocyte glutamate uptake was evaluated by the quantification of the clearance of exogenous glutamate (500 μM) from the astrocytes culture medium. Following 3 h of OGD insult, extracellular glutamate levels in the medium from cultured astrocytes with or without Rd pre-treatment (1, 10, and 50 μM, 12 h before OGD) were examined at different time intervals. 10 and 50 μM Rd significantly enhanced the glutamate uptake in astrocyte cultures, compared with the control (**Figure 1B**).

### Rd UP-REGULATES GLT-1 EXPRESSION AFTER MACO

**FIGURE 2:**
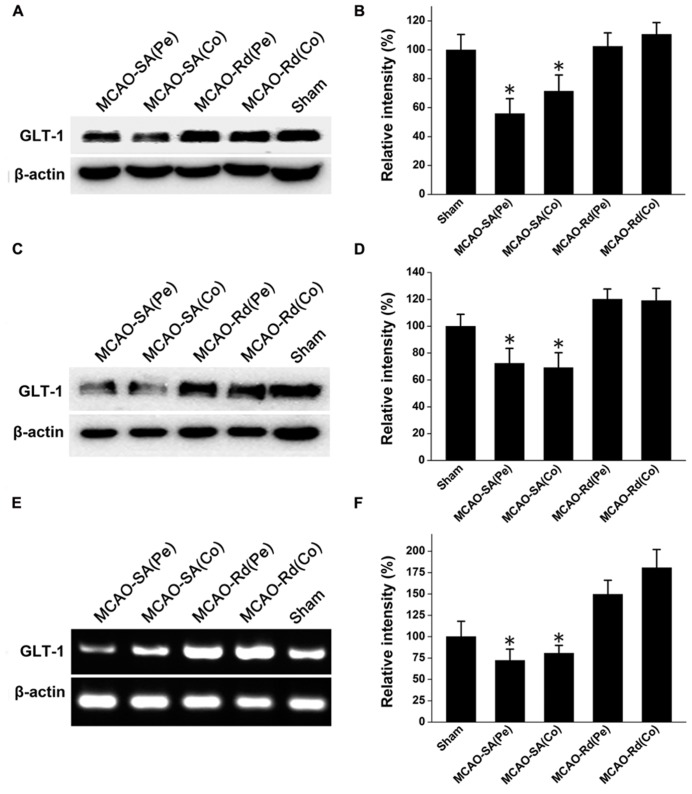
**Effect of Rd on GLT-1 expression in the core (Co) and penumbra (Pe) of ischemic brains. ** Western blotting analysis of GLT-1 expression at 4 h **(A,B)** and 24 h **(C,D)** following rat MCAO. **(E,F)** Analysis of relative GLT-1 mRNA expression by semi-quantitative RT-PCR 24 h after rat MCAO. Data are expressed as mean ± SEM (*n* = 9). Mean values in sham-treated groups were scaled to 100%. *p < 0.05 vs. Rd-treated group.

Since GLT-1 is one of main transporters for removal of extracellular glutamate accumulation, we next investigated whether Rd could affect GLT-1 expression during and after rat MCAO (**Figure 2**). Western blotting results showed that GLT-1 protein levels were reduced 4 and 24 h after MCAO in both core and penumbra of ischemic rat brains, consistent with previous reports (Chen et al., 2005; Han et al., 2008). Rd treatment up-regulated GLT-1 expression at both time points after rat MCAO (**Figures 2A–D**). Similarly, RT-PCR analysis also showed an increase in mRNA expression levels of GLT-1 in the presence of Rd 24 h after MACO (**Figures 2E,F**), suggesting that Rd up-regulates GLT-1 expression after MACO.

### Rd INCREASES THE LEVELS OFPHOSPHO-PI3K/AKT AND PHOSPHO-ERK1/2

**FIGURE 3:**
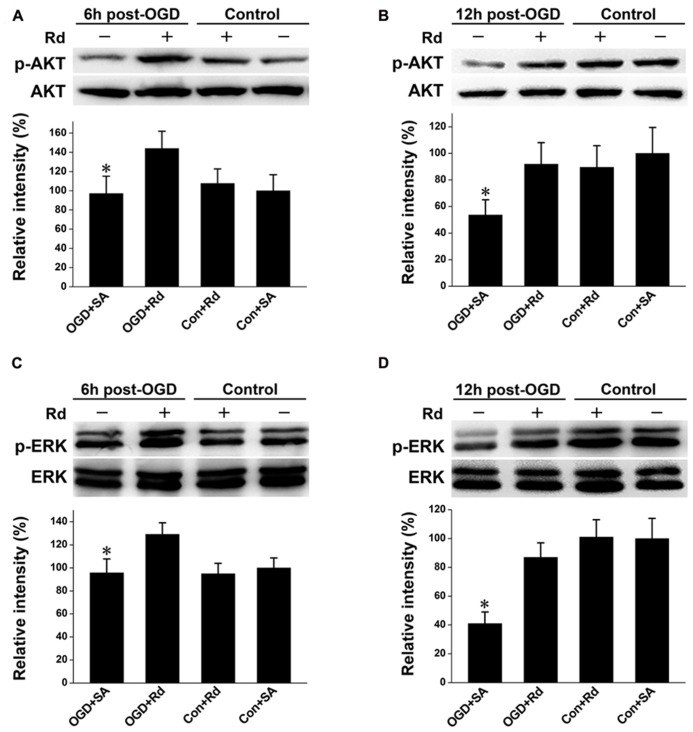
**Effect of Rd on phosphorylation of AKT and ERK1/2. ** Cultured astrocytes were harvested 6 and 12 h after OGD. Western blotting analysis of proteins extracted from astrocytes was performed using antibodies against *p*-AKT/AKT **(A**: 6 h and **B**: 12 h**)** or *p*-ERK1/2 **(C**: 6 h and **D**: 12 h**)** as described in Section “Materials and Methods.” Data are expressed as mean ± SEM (*n* = 6). Mean values in sham-treated groups were scaled to 100%. *p < 0.05 vs. OGD + Rd-treated group.

PI3K/AKT and ERK mediate crucial regulatory pathways of cell survival (Kumar et al., 2004; Liu et al., 2012a). Both pathways are known to induce GLT-1 expression (Li et al., 2006; Lee et al., 2012a). We then explored whether Rd-induced GLT-1 up-regulation was mediated through PI3K/AKT and/or ERK pathway in astrocyte culture subjected to OGD insult. Western blotting showed that the levels of phospho-AKT and phospho-ERK were reduced 6 and 12 h after OGD, consistent with previous reports (Liu et al., 2012a; Xie et al., 2012; Xu et al., 2012). After the treatment of Rd, the levels of phosphorylation of AKT and ERK were markedly enhanced (**Figure 3)**, indicating that Rd can activate PI3K/AKT and ERK pathways.

### PI3K/AKT AND ERK1/2 PATHWAYS ARE INVOLVED IN RD ENHANCED GLT-1 EXPRESSION AND GLUTAMATE UPTAKE

**FIGURE 4:**
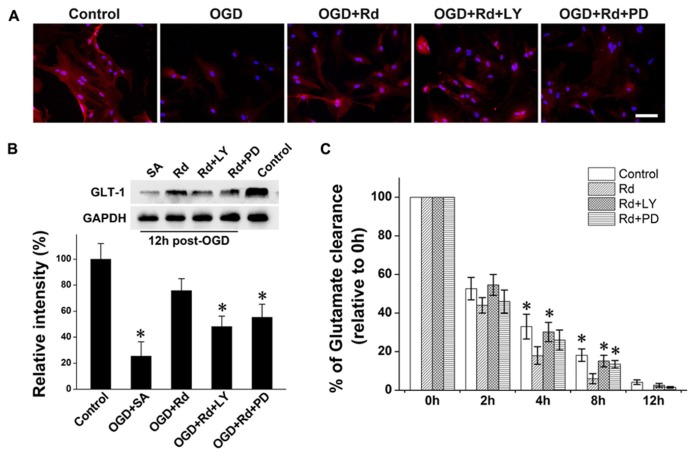
**PI3K/AKT and ERK are involved in Rd-induced GLT-1 up-regulation and glutamate uptake enhancement. ** 20 μM PD98059 (PD) or 5 μM LY294002 (LY) was pre-treated 12 h before OGD.** (A)** Double immunofluorescence labeling (GLT-Red, Hoechst-blue) 12 h after OGD. (**B)** Western blotting results 12 h after OGD. **(C)** Glutamate uptake assays. After 3 h of OGD, 500 μM glutamate was added to medium, and glutamate concentrations were measured at different time intervals (from 2 to 12 h after OGD washout). Data are expressed as mean ± SEM (*n*=6). Mean values in sham-treated groups were scaled to 100%. *p < 0.05 vs. OGD + Rd group.

Finally, we investigated whether Rd-induced GLT-1 up-regulation and glutamate uptake enhancement was mediated by PI3K/AKT and ERK1/2 pathways by using PI3K/AKT pathway inhibitor LY294002 or ERK1/2 pathway inhibitor PD98059. Western blotting and immunofluorescence labeling results showed that GLT-1 protein levels in cultured astrocytes decreased 12 h after OGD (**Figure 4A**). Similar to MCAO results, Rd increased GLT-1 expression 6 h (data not shown) and 12 h after OGD. While pre-treatment with LY294002 or PD98059 inhibited this effects of Rd (**Figure 4B**). Next, we tested the role of PI3K/AKT and ERK in Rd-induced glutamate uptake. As expected, Rd promoted astrocyte glutamate clearance, which can be abolished by co-treatment with LY294002 or PD98059 (**Figure 4C**). Taken together, these results suggest that Rd may promote GLT-1 expression and glutamate uptake through PI3K/AKT and ERK1/2 pathways.

## DISCUSSION

In the present study, we investigated the effects of Rd on the expression of the astrocytic glutamate transporter GLT-1 as well as glutamate uptake activity in cultured astrocytes and MCAO rat. The results showed that Rd increased mRNA and protein levels of GLT-1. This effects can be inhibited by the AKT or ERK1/2 inhibitor, suggesting that AKT and ERK1/2 signaling pathways are involved in Rd-mediated effects on extracellular glutamate metabolism.

Glutamate-induced excitotoxicity is an important factor responsible for cell death in many central nervous system (CNS) disorders (Benveniste et al., 1984). Excessive extracellular glutamate over-stimulates ionotropic GluRs, such as NMDA, subsequently initiating a series of downstream lethal events including oxidative stress, mitochondrial dysfunction, and inflammation (Szydlowska and Tymianski, 2010). Astrocytes play a crucial role in the removal of extracellular glutamate to prevent against glutamate excitotoxicity (Rothstein et al., 1996). Two types of glutamate transporters, GALST and GLT-1, are identified in astrocytes: GLAST is predominantly expressed in the cerebellum and required to keep extracellular glutamate at a physiological level (Watase et al., 1998). GLT-1 is abundant in various area of the forebrain, including hippocampus, cerebral cortex, and the striatum. It is responsible for more than 90% of glutamate uptake in these areas (Danbolt, 2001). A group of studies have showed that GLT-1 is required for uptake of extracellular glutamate to protect neurons under ischemic conditions. For example, knockdown of GLT-1 expression exacerbated ischemia-induced neuronal damage and causes enlarged infarct volume (Rao et al., 2001). In contrast, targeted over-expression of GLT-1 decreased the glutamate overflow and reduced the cellular and behavioral deficits induced by ischemic stroke (Harvey et al., 2011). Thus, GLT-1 plays a neuronal protective role during ischemic stroke. Several studies showed decreases in GLT-1 mRNA and protein levels in the rat brain following ischemic insult. In addition, reduction of GLT-1 expression was observed 6 h after cerebral ischemia and was in part causative of glutamate-induced neurotoxicity in early phases of cerebral ischemia (Yeh et al., 2005). In our study, administration of Rd significantly increased the expression of GLT-1 mRNA and protein levels. These results suggest that Rd alleviates excitatory toxicity at least in part by restoring the expression of GLT-1.

Additionally, GLT-1 is also proposed to release intracellular glutamate under ischemic conditions (Phillis et al., 2000). However, no effect of dihydrokainate, a blocker of the reversed GLT-1 uptake, on ischemia-induced glutamate release was observed (Roettger and Lipton, 1996). Glutamate release in less severely ischemic brain was shown to occur mainly *via* volume-activated anion channels but not via GLT-1 reversal (Feustel et al., 2004). These studies suggest that GLT-1 reversal may not be an essential mechanism for increased extracellular glutamate concentration, particularly in ischemic penumbra.

As critical mediators, AKT and ERK1/2 signaling pathways are involved in cell proliferation, differentiation, and adaptation (Kumar et al., 2004; Liu et al., 2012a). Phosphorylation of ERK1/2 and AKT activates the transcription factors, NF-kB, and cAMP-response element binding protein (CREB), which in turn control the transcription of GLT-1(Li et al., 2006; Lee et al., 2012a). In addition, other types of ginsenosides, such as Rb1, Rg1, have been reported to affect AKT and ERK1/2 pathways (Lan et al., 2011; Hashimoto et al., 2012; Yan et al., 2013). Our present study further showed that Rd activated AKT and ERK1/2 signaling pathways (**Figure 4**), subsequently affecting GLT-1 expression and extracellular glutamate uptake.

Estrogen receptor (ER)-mediated PI3K/AKT and ERK1/2 activation are well-documented in protection against cell death. Recently, several reports showed that ER activation increased GLT-1 expression and glutamate uptake (Lee et al., 2012a, b). Ginseng has been recommended for alleviation of the symptoms of menopause, indicating that some components of ginseng act as phytoestrogen involve activation of the ER (Amato et al., 2002). Some evidence showed that ginseng extracts were able to stimulate the growth of ER-positive cells (Duda et al., 1999). Ginsenosides were structurally and functionally similar to 17β-estradiol (Lee et al., 2003; Lau et al., 2008) and activated AKT and ERK pathways (Lan et al., 2011; Hashimoto et al., 2012; Yan et al., 2013). Thus, we proposed that acting on ER may account for Rd-induced AKT and ERK1/2 activation and sequential GLT-1 up-regulation. Yet, Rd may also affect the functions of GLT-1 in several ways, including cell trafficking, splicing, and post-translational modification. Thus, the possible links between Rd and ER still require further clarification.

In summary, in the present study we showed that Rd could promote extracellular glutamate clearance by up-regulating GLT-1 expression through PI3K/AKT and ERK1/2 pathways.

## Conflict of Interest Statement

The authors declare that the research was conducted in the absence of any commercial or financial relationships that could be construed as a potential conflict of interest.

## AUTHOR CONTRIBUTIONS

Xiao Zhang: most of the research work and paper writing. Ming Shi: part of the paper writing and experiment design. Magnar Bjørås: polishing manuscript, great assistance in main research project design. Wei Wang: paper revision, great assistance in experiment design. Guangyun Zhang: funds support. Junliang Han: great assistance in Microdialysis experiment. Zhirong Liu: great assistance in experiment design. Yunxia Zhang: doing part of the Western blot experiment. Bing Wang: doing part of the Immunocytochemistry experiment. Jing Chen: glial cultures and OGD experiment. Yi Zhu: doing the animal surgery. Lize Xiong: main research project design. Gang Zhao: main research project and experiment design.
